# Topical immunotherapy with diphenylcyclopropenone in paediatric patients with alopecia areata—A retrospective study of 97 patients

**DOI:** 10.1002/ski2.441

**Published:** 2024-08-19

**Authors:** Farzad Esmaeili, Seyed Mohammad Vahabi, Mohammadsadegh Abdoli, Patrick Fazeli, Narges Ghandi, Leila Seddigh, Zeinab Aryanian, Ifa Etesami

**Affiliations:** ^1^ School of Medicine Shahid Beheshti University of Medical Sciences Tehran Iran; ^2^ Department of Dermatology Razi Hospital, Tehran University of Medical Sciences Tehran Iran; ^3^ Division of Biology & Medicine Brown University Providence Rhode Island USA; ^4^ Department of Community Medicine Tehran University of Medical Sciences Tehran Iran

## Abstract

**Background:**

Alopecia areata (AA) is an autoimmune disease causing chronic non‐scarring hair loss. Different therapeutic regimens have been suggested for AA, which depend on patients' age, scalp involvement extent and duration. Topical immunotherapy with diphenylcyclopropenone (DPCP) is one of the treatment options for these patients.

**Objectives:**

We aimed to investigate the response to DPCP in paediatric AA patients.

**Methods:**

This retrospective study included 97 paediatric AA patients followed in the DPCP clinic from March 2016 to March 2021 at a referral dermatology hospital.

**Results:**

In a cohort of 97 paediatric patients with AA under treatment with DPCP, with a mean age of 11.10 ± 0.9, 53.6% of the patients were male. Patchy alopecia was the most prevalent type (45.4%). After 6 months of DPCP treatment, 51.5% showed no response, while 3.1% achieved complete response. At the 12‐month evaluation, among the 68 patients who continued treatment, complete response was observed in 8.8%. A significant positive correlation was found between alopecia type, specifically patchy, and treatment response (*p* = 0.031). Additionally, treatment duration emerged as a significant predictor of positive response at both six (OR 1.450, *p* = 0.026) and 12 months (OR 1.310, *p* = 0.043). A higher initial Severity of Alopecia Tool score was inversely correlated with treatment response (Spearman's rho −0.14, *p* = 0.002), indicating that initial disease severity may predict treatment efficacy.

**Conclusions:**

One year after the onset of DPCP in paediatric AA patients, the complete response and any hair regrowth rates were 8.8% and 61.8%, respectively. The milder initial disease severity and longer duration of treatment resulted in a better response.

## Graphical abstract

In a cohort of 97 paediatric patients with alopecia areata under treatment with DPCP, with a mean age of 11.10 ± 0.9, 53.6% of the patients were male. Patchy alopecia was the most prevalent type (45.4%). After 6 months of DPCP treatment, 51.5% showed no response, while 3.1% achieved complete response. At the 12‐month evaluation, among the 68 patients who continued treatment, complete response was observed in 8.8%. A significant positive correlation was found between alopecia type, specifically patchy, and treatment response (*p* = 0.031). Our study shows that a milder initial disease severity and a longer duration of treatment result in a better response. Alopecia type, specifically patchy type, could be a predictive factor for treatment response.

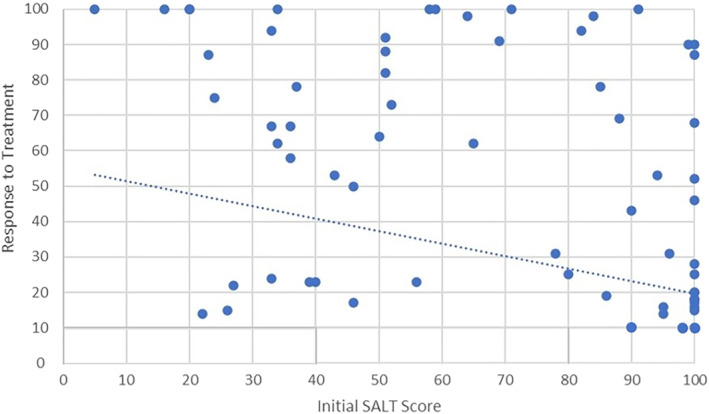

**What is already known?**

- Alopecia areata (AA) is an autoimmune disease causing non‐scarring hair loss. Different therapeutic regimens have been suggested for AA, and topical immunotherapy with diphenylcyclopropenone (DPCP) is one of them. Regrowth rates with DPCP vary from 33% to 83%. Previous studies have shown that multiple factors could affect the prognosis of patients, including the extent of scalp and body hair loss and a history of thyroid disorders.

**What does this study add?**

- Our study shows that a milder initial disease severity and a longer duration of treatment result in a better response. Alopecia type, specifically patchy type, could be a predictive factor for treatment response.

## 1 INTRODUCTION

Alopecia areata (AA) is an autoimmune disease causing chronic non‐scarring hair loss in patterns varying from diffuse or complete hair loss to well‐defined patches and potentially affecting all hair‐bearing sites.^1^ This autoimmune condition affects different aspects of one's quality of life beyond the mere hair loss, specifically in children afflicted by paediatric AA.^2^, ^3^ It is worth noting that a systematic review on eight comprehensive studies has shown that the overall quality of life in adolescents and children with AA is impaired in all domains, with embarrassment and self‐consciousness as the most consistently affected domains.^2^

Different therapeutic regimens have been suggested for AA, including intralesional and topical corticosteroids, psoralen and UVA (PUVA) phototherapy, anthralin, minoxidil and some newly introduced medications such as ruxolitinib, tofacitinib and baricitinib.^4^, ^5^, ^6^, ^7^ Particularly, for paediatric AA, the preferred first‐line treatment is topical corticosteroids.^8^ Janus kinase (JAK) inhibitors have also been recommended as efficacious therapies for AA.^5^ Among them, baricitinib and ritlecitinib are, respectively, the first FDA‐approved JAK inhibitors for AA^5^ and for paediatric AA cases aged 12 years and older.^9^

One of the alternative treatments for AA is the use of topical sensitizers, including diphenylcyclopropenone (DPCP), which can be used alone or in combination with other therapeutic methods.^10^, ^11^ Although it has been reported to be an effective topical immunotherapy for the treatment of extensive AA, the efficacy data are highly variable.^12^ Additionally, there are not many studies on the use of DPCP in children.

DPCP's precise mechanism of action is unknown, but it is theorized to reduce production of the anti‐hair‐follicle antibodies and induce antigenic competition.^13^, ^14^ Regrowth rates with DPCP vary from 33% to 83% averaging 50%.^12^, ^15^ Previous studies have shown that multiple factors could affect the prognosis of patients, including the extent of scalp and body hair loss and a history of thyroid disorders.^16^ In this study, we assess the response to DPCP in paediatric AA patients and investigate potential associated prognostic factors.

## 2 METHODOLOGY

### 2.1 Study design and setting

This study is a retrospective single‐centre observational study conducted at the alopecia (DPCP) clinic at a referral dermatology hospital. The study period spanned from March 2016 to March 2021, focusing on paediatric patients with a pathology‐confirmed diagnosis of AA.

### 2.2 Participants

**FIGURE 1:**
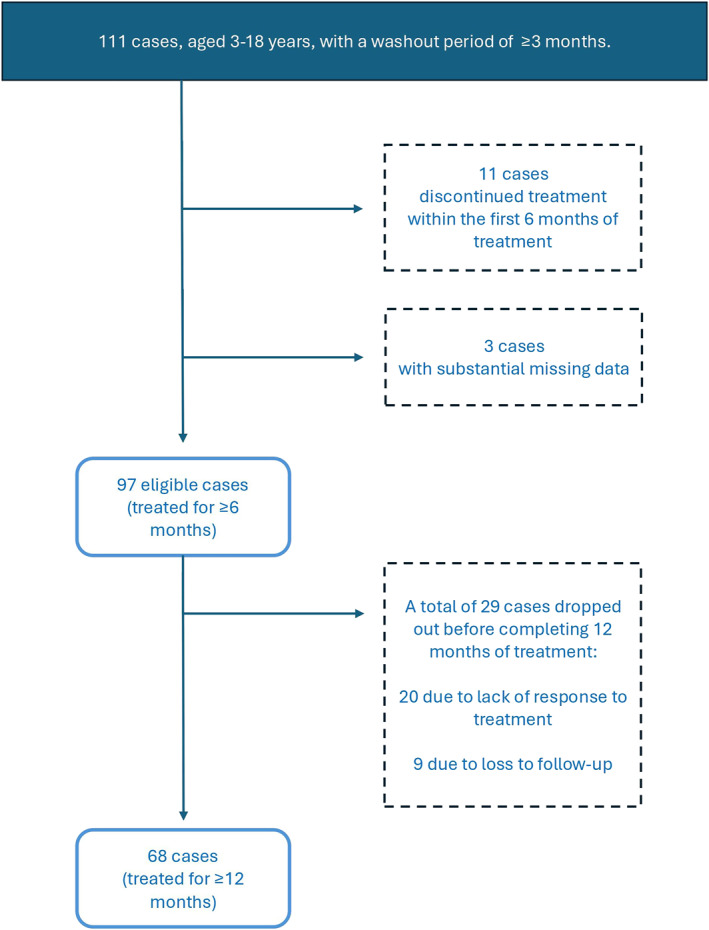
Flow chart demonstrating the inclusion and exclusion of patients.

Initially, a total of 111 paediatric AA patients, aged 3–18 years were considered. It is worth noting that for the inclusion criteria we essentially considered and included only the patients who had not received any kind of topical or systemic treatment for at least 3 months prior to the onset of DPCP treatment. Accordingly, the washout period in our study was established to be 3 months (Figure 1). Therefore, we ascertained that the patients included were not receiving any concurrent treatments. Additionally, this study primarily assessed AA on the scalp, excluding other areas. Further, patients who discontinued treatment within 6 months of onset (*n* = 11), and those with substantial missing data in their electronic health records (*n* = 3) were excluded (Figure 1). This meant that ultimately 97 eligible paediatric AA cases, aged 3–18 years, with no substantial missing data and with a minimum DPCP therapy duration of 6 months were included. We rigorously tracked participants throughout the study primarily because we observed a number of patients discontinuing treatment. The main reason was a lack of effective response, which led to discontinuation in 20 patients, and further 9 patients were reluctant to continue with the treatment due to the considerable distance between their residences and the treatment centre (loss to follow‐up) (Figure 1). This study has been approved by the ethics institutional review board of Tehran University of Medical Sciences, Tehran, Iran (Ethical code: IR.TUMS.MEDICINE.REC.1398.161). Written informed consent was obtained from the patients' parents or legal guardians.

### 2.3 Data sources, data collection and measurements

Patient data, including demographic characteristics, medical history and clinical course during treatment were meticulously extracted from hospital medical records, with independent verification performed by medical researchers. The treatment process was initiated by sensitizing patients with a 2% concentration of DPCP in acetone applied to a 4 × 4 cm area on one hemisphere of the scalp, using a cotton applicator. After 2 weeks, if the patients were responsive to DPCP, the treatment would continue on the same area with a 0.001%–0.01% concentration.^17^ Treatments continued weekly, and the DPCP concentration was gradually increased to 2% to induce mild contact dermatitis. The concentration was adjusted based on dermatitis severity, increased if symptoms lessened and decreased if blistering occurred or treatment was delayed. Physicians closely monitored patients' responses and adjusted treatments accordingly. As for the follow‐up frequency, the patients were visited weekly for the first 4 weeks of treatment, then monthly until hair regrowth was seen and eventually every 2–3 months. The rationale behind it was rooted in the need for precise dose adjustment and monitoring of treatment response and side effects, especially for patients during the early phases of therapy. This aligns with dermatological guidelines, which emphasize that close observation in the early stages of treatment enhances success rates and patient compliance.^17^, ^18^, ^19^

The Severity of Alopecia Tool (SALT) score served as the primary measure of treatment efficacy. SALT scores were recorded at the initiation of treatment, and then at the sixth‐ and twelfth‐month mark. The percentage change in hair growth was determined using the formula given below:

$$\frac{\text{Initial}\;\text{SALT}\;\text{Score}-\text{Final}\;\text{SALT}\;\text{Score}}{\text{Initial}\;\text{SALT}\;\text{Score}}\times 100$$

This calculation categorized hair growth into four groups: no response (0%–10%), minimal response (11%–50%), partial response (51%–90%) and complete response (91%–100%). A patient demonstrating at least 10% hair regrowth was classified as a treatment responder. This categorization aligns with those commonly used in similar studies, such as those conducted by Ohlmeier et al., Luk et al. and Salsberg et al.^20^, ^21^, ^22^

### 2.4 Statistical analysis

The statistical analysis was structured to provide a comprehensive overview of patient characteristics and factors influencing the treatment response. Initially, descriptive statistics were employed to summarize the demographic and clinical characteristics of the paediatric patients. This involved calculating frequencies and percentages for categorical variables such as sex, type of AA and initial site of hair loss. To investigate the correlation between the response to treatment and potential prognostic factors, chi‐square tests or Fisher's exact tests were applied for categorical variables (i.e. age, sex, type and site of AA, nail involvement and prior treatment history), and *t*‐tests or ANOVA for continuous variables (i.e. duration of treatment). These analyses facilitated the evaluation of associations between treatment outcomes at 6 and 12 months (categorized as no response, partial response, moderate response and complete response) and various factors such as age, sex, type of alopecia and duration of treatment. Furthermore, logistic regression analysis was employed to explore the causal impact of prognostic factors on the treatment response, specifically those prognosticators previously yielding statistically significant results in the preliminary correlation analysis. This involved investigating alopecia type and treatment duration in terms of calculating odds ratios, confidence intervals and *p*‐values, providing insights into the likelihood of treatment effectiveness based on those variables. To evaluate the correlation between the initial SALT score and the patient's response to treatment at the 12‐month mark, Spearman's rank correlation coefficient was used. This non‐parametric measure was chosen due to its ability to identify monotonic relationships, whether linear or not, between two continuous or ordinal variables. The strength and direction of the correlation were determined by the correlation coefficient, with values ranging from −1 to +1, where +1 indicates a perfect positive correlation, 0 indicates no correlation and −1 indicates a perfect negative correlation. Statistical significance was set at a two‐sided alpha of <0.05. All analyses were conducted using SPSS 25.0 (SPSS).

## 3 RESULTS

The study encompassed 97 patients with AA, with a slight male predominance of 53.6%. The mean age was 11.10 ± 0.9, and the age distribution was notably higher in the 6–12 years range, accounting for 43.3% of the patient cohort. It is worth noting that to enhance the clarity and depth of our demographic data, additional variables such as familial history of alopecia and background diseases have been included in Table 1. This comprehensive demographic overview aids in assessing the representativeness of our study cohort and provides insights into potential confounding factors that could influence treatment outcomes. A familial history of alopecia was present in 14.4% of cases, and the majority (87.6%) had no background diseases. The most common alopecia type was patchy (45.4%), followed by universalis (28.9%), with the occipital area being the most common initial site of hair loss in 51.5% of patients. Only 9.3% of patients showed nail involvement, and prior treatments majorly included topical steroids (58.1%) and minoxidil (27.4%), with other less‐common treatments (Table 1).

**TABLE 1:** Initial evaluation of demographical and clinical characteristics of patients.

	Number of patients (valid percent)
Sex	
Male	52 (53.6%)
Female	45 (46.4%)
Age	
<6 y.o.	18 (18.6%)
6–12 y.o.	42 (43.3%)
12–18 y.o.	37 (38.1%)
History of familial alopecia	
Present	14 (14.4%)
Absent	83 (85.6%)
History of background diseases	
Thyroid disease	4 (4.1%)
Asthma	1 (1.0%)
Allergic rhinitis	5 (5.1%)
Atopic dermatitis	2 (2.1%)
No disease	85 (87.6%)
Type of alopecia	
Universalis	28 (28.9%)
Patchy	44 (45.4%)
Totalis	19 (19.6%)
Ophiasis	6 (6.2%)
Initial site of hair loss	
Occipital	50 (51.5%)
Parietal	21 (21.6%)
Frontal	11 (11.3%)
Vertex	15 (15.5%)
Nail involvement	
Present	9 (9.3%)
Absent	88 (90.7%)
Prior treatments	
Topical steroids	36 (58.1%)
Systemic steroids	7 (11.3%)
Minoxidil	17 (27.4%)
Anthralin	2 (3.2%)
Not recorded	35

After 6 months of DPCP treatment, 50 (51.5%) of the 97 patients showed no response, while 30 (30.9%) exhibited a minimal response, 14 (14.4%) had a partial response and 3 (3.1%) achieved a complete response. Progressing to the 12‐month interval, among the 68 patients who continued treatment, the response rates varied with 26 (38.2%) patients showing no response, 18 (26.5%) minimal response, 18 (26.5%) partial response and 6 (8.8%) demonstrating complete response. This progression suggested an increasing trend of positive response with extended treatment duration (Table S1).

The study found a significant correlation between the type of AA and treatment response at 6 months. Patients with patchy alopecia responded more favourably compared to those with other types, as evidenced by the statistical significance (*p* = 0.031) (Table 2). In addition, the findings suggested that the duration of disease had a significant correlation with treatment response. Patients who did not respond to treatment had a significantly longer duration of disease recorded (12.5 vs. 1.9 years, *p* = 0.049), suggesting that earlier interventions may lead to better treatment outcomes (Table 2). However, this association was attenuated to insignificance after completing 12 months of treatment (*p* = 0.092) (Table 3). Further, the duration of treatment prominently influenced treatment response, both at 6 and 12 months, with statistically significant values (*p* = 0.001 at both intervals). At 6 months of treatment patients responding to treatment had a longer average treatment duration compared to non‐responders (15.4 vs. 12.1 months, *p* = 0.001). Moreover, after 12 months of treatment, responders had a longer average treatment duration than non‐responders (19.4 vs. 13.2 months, *p* = 0.001) (Table 3). However, other factors such as age, initial site of hair loss and history of atopic disease did not exhibit a significant impact on the treatment outcome. In addition to the statistical comparison between responders and non‐responders, we compared the patients with <50% response rate and those with more than 50% response rate. The findings demonstrated that after 6 months of treatment the only variable that showed significant difference between the two groups, was duration of treatment which was longer in patients with more than 50% response rate (13.4 vs. 14.9 months, *p* = 0.048). Similarly, after completing 12 months of treatment, duration of treatment was still the only variable among all potential prognostic factors that harboured significant difference between the two groups (14.6 vs. 17.2 months, *p* = 0.021) (Table S2).

**TABLE 2:** Correlation between the response to treatment and potential prognostic factors in all 97 patients completing 6 months of treatment.

All patients	Non‐responders	Responders			*p*‐value
	No response	Minimal response	Partial response	Complete response	
	(*n* = 50)	(*n* = 30)	(*n* = 14)	(*n* = 3)	
Sex					0.243
Male	27 (54.0%)	25 (53.2%)			
Female	23 (46.0%)	22 (46.80%)			
Age groups					0.730
<6 y.o.	10 (20.0%)	8 (17.0%)			
6–12 y.o.	23 (46.0%)	19 (40.4%)			
12–18 y.o.	17 (34.0%)	20 (42.5%)			
Alopecia type					0.031*
Universalis	16 (32.0%)	12 (25.5%)			
Patchy	20 (40.0%)	24 (51.1%)			
Totalis	11 (22.0%)	8 (17.0%)			
Ophiasis	3 (6.0%)	3 (6.4%)			
Initial site of hair loss					0.493
Occipital	26 (52.0%)	24 (51.1%)			
Parietal	11 (22.0%)	10 (21.3%)			
Frontal	6 (12.0%)	5 (10.6%)			
Vortex	7 (14.0%)	8 (17.0%)			
Nail involvement					0.751
Present	5 (10.0%)	4 (8.5%)			
Absent	45 (90.0%)	43 (91.5%)			
History of atopic disease					0.386
Present	7 (14.0%)	7 (14.9%)			
Absent	43 (86.0%)	40 (85.1%)			
Duration of treatment in months (mean ± SD)	12.1 ± 1.2	15.4 ± 3.8			0.001*
Duration of disease in years (mean ± SD)	2.5 ± 1.3	1.9 ± 0.9			0.049*

*Note*: Bold values represented *p* value < 0.05 which means a statistically significant relationship.

**TABLE 3:** Correlation between the response to treatment and potential prognostic factors in all 68 patients completing 12 months of treatment.

All patients	Non‐responders	Responders			*p*‐value
	No response	Minimal response	Partial response	Complete response	
	(*n* = 26)	(*n* = 18)	(*n* = 18)	(*n* = 6)	
Sex					0.199
Male	4 (15.4%)	9 (21.4%)			
Female	22 (84.6%)	33 (78.6%)			
Age groups					0.780
<6 y.o.	6 (23.1%)	10 (23.8%)			
6–12 y.o.	10 (38.5%)	13 (30.9%)			
12–18 y.o.	10 (38.5%)	19 (45.2%)			
Alopecia type					0.054
Universalis	8 (30.8%)	12 (28.6%)			
Patchy	13 (50.0%)	20 (47.6%)			
Totalis	2 (7.7%)	7 (16.7%)			
Ophiasis	3 (11.5%)	3 (7.1%)			
Initial site of hair loss					0.059
Occipital	9 (34.6%)	19 (45.2%)			
Parietal	8 (30.7%)	11 (26.2%)			
Frontal	5 (19.2%)	3 (7.1%)			
Vortex	4 (15.4%)	9 (21.4%)			
Nail involvement					0.644
Present	4 (15.4%)	7 (16.7%)			
Absent	22 (84.6%)	35 (83.3%)			
History of atopic disease					0.401
Present	3 (11.5%)	5 (11.9%)			
Absent	23 (88.5%)	37 (88.1%)			
Duration of treatment in months (mean ± SD)	13.2 ± 8.9	19.4 ± 9.1			0.001*
Duration of disease in years (mean ± SD)	2.4 ± 1.1	2.3 ± 0.9			0.092

*Note*: Bold values represented *p* value < 0.05 which means a statistically significant relationship.

The logistic regression analysis demonstrated that at both the 6‐ and 12‐month interval, the duration of treatment in the clinic significantly affected the response to treatment (6‐month mark: OR 1.450, *p* = 0.026; 12‐month mark: OR 1.310, *p* = 0.043). This finding reinforced the notion that a longer duration of DPCP treatment is likely to result in more favourable outcomes. However, the AA type did not show a significant impact on treatment response in the regression analysis (Table 4).

**TABLE 4:** Logistic regression analysis on the response to treatment based on potential prognostic factors.

	At 6‐month mark		At 12‐month mark	
	Odds ratio (95% confidence interval)	*p*‐value	Odds ratio (95% confidence interval)	*p*‐value
Alopecia types	0.284 (0.087–1.813)	0.644	‐	‐
Duration of treatment in the clinic	1.450 (1.117–2.216)	0.026*	1.310 (1.106–2.922)	0.043*

*Note*: Bold values represented *p* value < 0.05 which means a statistically significant relationship.

**FIGURE 2:**
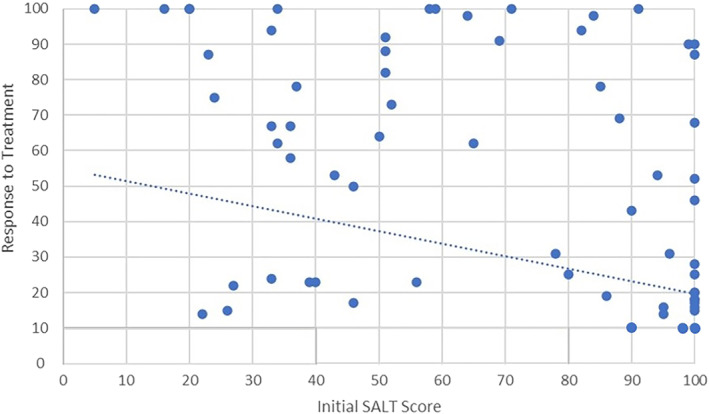
Correlation between response to treatment and the initial Severity of Alopecia Tool score.

The Spearman's rank correlation coefficient was used to evaluate the correlation between the initial SALT score and the patient's response to treatment. The correlation analysis between the initial SALT score and the 12‐month mark response to treatment revealed a statistically significant inverse relationship, as evidenced by a Spearman's rho correlation coefficient of −0.14 (*p* = 0.002). This suggests that a higher initial SALT score, indicative of more severe AA, is associated with a lower likelihood of a positive response to DPCP treatment over the course of a year. The analysis underscores the initial severity of AA as a potential predictor of treatment efficacy (Figure 2).

Furthermore, we assessed the complications reported for patients in our study (Table S3). After 6 months of treatment, 52 cases (53.6%) develop no complications, with blisters and vesicles as the common complication manifested in 27 cases (38.1%). Importantly, no statistically significant association was found between response to treatment and any of the complications. Furthermore, after 12 months of treatment, 38 cases (55.9%) had developed complications with blister and vesicles still as the most common complication observed in 25 patients (36.8%). No statistically significant difference was found between the responders and non‐responders in terms of complications. It is also worth mentioning that urticaria as a commonly occurring dermatologic complication, was not found in any of the patients.

## 4 DISCUSSION

This study investigated the response to DPCP treatment in paediatric patients with AA at a referral dermatology hospital, assessing potential prognostic factors. Our analysis revealed that for those completing the 12‐month treatment period, the partial and complete response rates for DPCP treatment were 26.5% and 8.8%, respectively. The overall percentage of responders increased from 48.5% at 6 months of treatment to 61.8% at 12 months of treatment, indicating that a significant proportion of patients experienced positive response with extended treatment duration.

Our findings advocate for a more integrated role of DPCP in the management of paediatric AA, aligning with current clinical guidelines that typically reserve DPCP as a secondary treatment. The data suggest that DPCP is effective for paediatric mild to moderate patients unresponsive to first‐line treatments, including topical corticosteroids. Although JAK inhibitors have been recently shown to be more effective than DPCP, their use in children is not FDA‐approved and they may pose side effects. Given the chronic nature of AA and its psychological impact, initiating DPCP earlier could be beneficial, especially in cases of mild AA. The correlation between lower initial SALT scores and better outcomes with DPCP supports its potential utility not only as a secondary option but also as a primary consideration in certain clinical situations.

Other studies have investigated the use of DPCP as a topical sensitizer in the treatment of paediatric AA. In a prospective study by Schuttelaar et al., among the 25 AA cases, 84% of the children showed any hair regrowth after DPCP therapy. While 32% of the cases had cosmetically acceptable hair regrowth.^23^ In a Chinese study by Luk et al., 31 AA patients with a mean age of 17.8 years were retrospectively evaluated after receiving DPCP treatment. 37.9% of these cases achieved either partial regrowth or complete regrowth, which was somehow comparable to our analysis results; at 12 months, 35.3% of our cases were recorded to have either partial response or complete response.^20^

In a more large‐scale analysis performed by Salsberg et al. on 108 paediatric AA patients aged from 4 months to 18 years, 13% of cases showed complete hair regrowth after 6 months of treatment, and 25% showed partial regrowth; whereas, in our study, at 6 months of treatment, 3.1% and 14.4% showed complete and partial hair regrowth, respectively. Moreover, at 12 months of treatment, 11% of cases showed complete hair regrowth and 21% showed partial regrowth. None of the known prognostic factors, including the nail involvement, family history, onset age, coexisting autoimmune disorder, atopy and extent of involvement was related to a greater possibility of having a complete or partial response to DPCP treatment.^22^ Furthermore, in another large‐scale study by Ohlmeier et al. on 142 AA patients, 37.8% of patients had a complete response and 14.8% exhibited a partial response. Additionally, in line with our findings, Ohlmeier et al. showed that the severity of disease and subsequently the extent of hair loss prior to treatment were the major predictive factor for the therapeutic success of DPCP.^21^

In fact, the study's findings are in line with previous research, although our response rates were slightly lower than some other studies. This discrepancy could be attributed to differences in patient demographics (specifically age and ethnicity), the severity of the disease at onset and the types of AA prevalent in our patient population. Our results underscored the importance of duration of treatment as well as the initial severity of the disease as significant factors influencing treatment outcomes. Particularly, patients with a higher initial SALT score showed lower response rates, confirming the negative impact of severe disease at the onset of treatment. Accordingly, we have demonstrated that as the initial SALT score of patients increased, the response rate to treatment decreased, so the initial severity of the disease and the pre‐treatment extent of hair loss could be deemed as prominent prognostic factors, as evidently reflected in a similar study on AA paediatric patients.^23^ Although we observed correlations between treatment response and factors such as alopecia type and treatment duration using logistic regression, these findings should be interpreted with caution due to the modest response rates. Larger studies are necessary to confirm these predictors.

A notable observation was the impact of AA type on treatment response, with universalis and totalis types being more prevalent among non‐responders, highlighting the variable efficacy of DPCP across different AA types. The potential effect of different types of AA has been confirmed by the findings in several studies^15^, ^24^, ^25^, ^26^, ^27^; while, in others, no effect for this parameter could be found.^28^, ^29^ Regularity and duration of clinic visits also emerged as significant predictors of treatment success. This finding suggests the importance of patient adherence to treatment schedules, although it is essential to consider that regular clinic visits could also be a consequence of successful treatment, prompting continued engagement from patients experiencing positive outcomes. Those with an inherently better response to treatment are clearly more eager and determined to refer to the clinic on a more regular basis; on the contrary, those who did not get a favourable response from the treatment (regardless of the reason) were more likely to discontinue the treatment and stop referring to the clinic. Long‐term patient compliance is critical for achieving better outcomes with DPCP treatment. Strategies to improve adherence, especially in paediatric populations, are essential and should be a focus of future studies.

Comparisons with adult studies on DPCP treatment suggest that response rates might differ between paediatric and adult patients. Studies on adult populations have generally reported higher response rates, indicating possible age‐related variations in DPCP efficacy. In a study by Nasimi et al. conducted on adult AA patients with a mean age of 25 years, 50%–90% regrowth rate and complete response rates were found to be 31.7% and 16.3%, respectively, both evidently >26.5% and 8.8% which was observed in our study for children.^30^ In fact, given the modest response rates observed with DPCP, it is suggested that DPCP be considered as a second‐line therapy, particularly for adult patients who do not respond to topical corticosteroids. However, our findings propose that DPCP would be effective for paediatric patients with mild to moderate AA who do not respond to usual first‐line treatments (including topical corticosteroids). Although JAK inhibitors have recently shown higher efficacy, their use in children is still not FDA‐approved and may pose significant side effects. Another study conducted by Ohlmeier et al. on adult AA patients with a mean disease onset age of 30 years showed that in over 50% of patients, the hair regrowth rate was 53%.^21^ Moreover, in accordance with another study by Zerbinati et al. on adult AA patients with a mean disease onset age of 35 years, the overall response rate to DPCP treatment was found to be 44.05%.^31^ More studies are indeed required to shed further light on how this difference in treatment response could be explained both clinically and etiologically.

In assessing the tolerability of DPCP, it is crucial to recognize that while side effects such as blisters and vesicular eruptions are not uncommon, they are generally mild and manageable with proper care. In our study, after 6 months of treatment, 53.6% of cases had no complications, while 38.1% experienced blisters and vesicles. After 12 months, 55.9% of patients developed complications, with blisters and vesicles remaining the most common in 36.8% of patients. In real‐world settings, this entails regular monitoring and timely intervention to adjust treatment concentrations to minimize discomfort. Our findings highlight the importance of careful patient selection and regular monitoring to manage common complications such as blisters and vesicles effectively. Educating patients and their families about potential side effects and their management is crucial to ensure adherence and optimize treatment outcomes. Education of parents and guardians about potential side effects and their management is also essential to ensure adherence and set realistic expectations about the treatment process. Moreover, the psychological benefits of successful treatment outcomes were often considered to outweigh the transient physical side effects. This aspect highlights the need for a holistic approach to treatment planning in paediatric AA, where psychological and physical considerations are integrated to optimize therapeutic success and patient well‐being.

Although our study only assessed the efficacy of DPCP monotherapy, the role of combination therapies including DPCP should not be overlooked. Particularly, studies have shown the effectiveness of combining DPCP with imiquimod for the treatment of AA. A review study by Mahasaksiri et al. reported that DPCP when combined with imiquimod, has evident superior efficacy compared to DPCP monotherapy.^32^ Furthermore, a case series by Díaz‐Guimaraens et al. has suggested that imiquimod‐enhanced immunotherapy with DPCP could be a promising way of improving the efficacy of contact immunotherapy in AA patients.^33^ Recently, the use of DPCP as a preferred AA treatment option has been debated. Currently, based on the most recent studies, the recommended prioritized place of DPCP for AA treatment might be prone to being dethroned by JAK inhibitors.^34^, ^35^ However, it must be noted that the use of JAK inhibitors for AA treatment has been FDA‐approved only for paediatric cases with above 12 years of age, namely Upadacitinib.^36^ In addition, there are certain important contraindications and complications^35^, ^37^ to investigate when considering JAK inhibitors for the treatment of paediatric AA patients; for instance, their use in patients with existing cardiovascular conditions^38^ and malignancies^37^ are contraindicated because they might potentially aggravate these conditions and could be deemed as life‐threatening. Therefore, many physicians still deem DPCP as a favourable treatment option for paediatric cases. Our study confirmed these recommendations by showing that for paediatric AA cases regardless of age, DPCP could be a safe and efficiently beneficial therapeutic option, with little to no serious life‐threatening complications and contraindications, specifically if received for at least 12 months. Although, emerging treatments such as JAK inhibitors have shown higher efficacy in treating AA, particularly in a non‐paediatric case, future research should focus on comparative studies to establish the optimal treatment hierarchy for paediatric AA, considering both efficacy and safety profiles.

The limitations of our study are as follows: First, since AA patients receiving DPCP therapy from Razi hospital were included, our study could be susceptible to selection bias. In fact, this sample of patients might not have necessarily reflected the population of all AA patients. While our sample size of 97 patients is reasonable, it may be insufficient to detect smaller effect sizes or rare side effects. Additionally, the single‐centre nature of our study limits the generalizability of our findings. Larger, multi‐centre studies are needed to validate our results. As the case selection was not randomized in this study, to avoid selection bias, we attempted to make the study groups as comparable as possible by matching the variables. Second, we must take into account that the regular clinic visits could also be a consequence of successful treatment rather than a cause, prompting continued engagement from patients experiencing favourable outcomes and discontinued engagement from patients with adverse outcomes. As a solution to this issue, conducting further randomized clinical trials on this topic could be suggested. Third, our study was retrospective in nature, which practically precludes definitive conclusions about causality of the associations. In fact, this retrospective nature introduces potential biases and limits our ability to control for confounding variables. Prospective, randomized controlled trials are needed to provide stronger evidence of the efficacy and safety of DPCP in paediatric AA. Fourth, the precise data on the administration of certain diagnostic and monitoring measures such as trichoscopy was lacking in this study. Lastly, our study did not assess the clinical manifestations of the disease in areas other than the scalp, such as eyebrows, eyelashes and body hair. Therefore, the effectiveness of DPCP on areas other than the scalp has not been particularly evaluated in this study. Of course, further comprehensive studies are needed to shed more light on the matter.

Future investigations should prioritize direct comparisons of DPCP with other treatments, such as JAK inhibitors and combination therapies, in paediatric AA. Long‐term longitudinal studies are essential to evaluate the persistence and durability of treatment response and the possibility of relapse after stopping treatment. Moreover, integrating patient‐ and parent‐reported outcomes will offer a more complete and a more holistic understanding of DPCP's impact, specifically given the psychological burden of the AA on children.

## 5 CONCLUSION

Our study provides valuable insights into the efficacy of DPCP treatment in paediatric AA patients by showing the complete and any hair regrowth rates to be 8.8% and 61.8%, respectively; after at least 12 months of treatment, it highlights the importance of early disease severity, AA type and treatment adherence as key factors influencing treatment outcomes. The results underscore the need for personalized treatment approaches and continuous patient engagement to optimize therapeutic success.

## CONFLICT OF INTEREST STATEMENT

None to declare.

## AUTHOR CONTRIBUTIONS

**Farzad Esmaeili**: Data curation (equal); formal analysis (lead); methodology (equal); writing – original draft (lead). **Seyed Mohammad Vahabi**: Formal analysis (equal); writing – original draft (equal); writing – review & editing (lead). **Mohammadsadegh Abdoli**: Conceptualization (equal); data curation (equal); methodology (supporting); project administration (equal); writing – original draft (equal). **Patrick Fazeli**: Formal analysis (equal); writing – review & editing (equal). **Narges Ghandi**: Conceptualization (equal); investigation (equal); validation (equal). **Leila Seddigh**: Conceptualization (equal); investigation (equal); validation (equal). **Zeinab Aryanian**: Methodology (equal); visualization (equal). **Ifa Etesami**: Conceptualization (lead); project administration (lead); supervision (lead); validation (supporting); writing – review & editing (supporting).

## ETHICS STATEMENT

This study has been approved by the ethics institutional review board of Tehran University of Medical Sciences, Tehran, Iran (Ethical code: IR.TUMS.MEDICINE.REC.1398.161).

## PATIENT CONSENT

Written informed consent was obtained from the patients' parents or legal guardians.

## Supporting information

Tables S1–S3

## Data Availability

The data underlying this article will be shared with the corresponding author on reasonable request.
