# Supplementary material for: Topical immunotherapy with diphenylcyclopropenone in paediatric patients with alopecia areata—A retrospective study of 97 patients
Source: Skin Health Dis. 2024 Aug 19;4(5):e441. doi: 10.1002/ski2.441 (PMC11442043; doi:10.1002/ski2.441)
Supplement: Supplementary file 1 — Tables S1–S3 [file SKI2-4-e441-s001.docx]

| **Supplementary Table 1.** Response to treatment in patients after 6 and 12 months of treatment | |
| --- | --- |
|  | **Number of Patients**  ***(Percent Out of remaining patients after dropout)*** |
| **After 6 months of treatment** (97 Patients) |  |
| - **No Response** | 50 (51.5%) |
| - **Minimal Response** | 30 (30.9%) |
| - **Partial Response** | 14 (14.4%) |
| - **Complete Response** | 3 (3.1%) |
| **After 12 months of treatment** (68 Patients) |  |
| - **No Response** | 26 (38.2%) |
| - **Minimal Response** | 18 (26.5%) |
| - **Partial Response** | 18 (26.5%) |
| - **Complete Response** | 6 (8.8%) |

| **Supplementary Table 2.** Correlation between the response to treatment and potential prognostic factors in all patients completing 6 and 12 months of treatment | | | | | | |
| --- | --- | --- | --- | --- | --- | --- |
| **All patients** | **After 6 months of treatment** | | | **After 12 months of treatment** | | |
|  | Less than 50% response | more than 50% response | P-value | Less than 50% response | more than 50% response | P-value |
|  | n=80 | n=17 |  | n=44 | n=24 |  |
| **Sex** |  | | 0.791 |  | | 0.096 |
| - **Male** | 43 (53.7%) | 9 (52.9%) |  | 7 (15.9%) | 6 (25.0%) |  |
| - **Female** | 37 (46.3%) | 8 (47.1%) |  | 37 (84.1%) | 18 (84.1%) |  |
| **Age Groups** |  | | 0.328 |  | | 0.063 |
| - **< 6 y. o** | 14 (17.6%) | 4 (23.5%) |  | 11 (25.0%) | 5 (20.8%) |  |
| - **6-12 y. o** | 35 (43.7%) | 7 (41.2%) |  | 16 (36.4%) | 7 (29.2%) |  |
| - **12-18 y. o** | 31 (38.7%) | 6 (35.3%) |  | 17 (38.6%) | 12 (50.0%) |  |
| **Alopecia Type** |  | | 0.476 |  | | 0.841 |
| - **Universalis** | 23 (28.7%) | 5 (23.5%) |  | 13 (29.5%) | 7 (29.2%) |  |
| - **Patchy** | 36 (45.0%) | 8 (41.2%) |  | 21 (47.7%) | 12 (50.0%) |  |
| - **Totalis** | 14 (18.7%) | 3 (29.4%) |  | 7 (15.9%) | 2 (8.3%) |  |
| - **Ophiasis** | 5 (7.5%) | 1 (5.9%) |  | 3 (6.8%) | 3 (12.5%) |  |
| **Initial Site of Hair Loss** | | | 0.663 |  | | 0.053 |
| - **Occipital** | 41 (51.2%) | 9 (52.9%) |  | 20 (45.4%) | 8 (33.3%) |  |
| - **Parietal** | 18 (22.5%) | 3 (17.6%) |  | 11 (25.0%) | 8 (33.4%) |  |
| - **Frontal** | 9 (11.25%) | 2 (11.8%) |  | 3 (6.8%) | 5 (20.8%) |  |
| - **Vortex** | 12 (15.0%) | 3 (17.6%) |  | 10 (22.7%) | 3 (12.5%) |  |
| **Nail Involvement** |  | | 0.128 |  | | 0.644 |
| - **Present** | 8 (10.0%) | 1 (5.8%) |  | 8 (18.2%) | 3 (12.5%) |  |
| - **Absent** | 72 (90.0%) | 16 (94.1%) |  | 36 (81.8%) | 21 (87.5%) |  |
| **History of Atopic Disease** | |  | 0.564 |  |  | 0.401 |
| - **Present** | 12 (15.0%) | 2 (11.8%) |  | 7 (15.9%) | 4 (16.7%) |  |
| - **Absent** | 68 (85.0%) | 15 (88.2%) |  | 37 (84.1%) | 20 (83.3%) |  |
| **Duration of Treatment**  **In months (mean ± SD)** | 13.4 ± 1.8 | 14.9 ± 2.9 | **0.048*** | 14.6 ± 7.1 | 17.2 ± 6.5 | **0.021*** |
| **Duration of Disease in years (mean ± SD)** | 2.4 ± 1.2 | 2.1 ± 1.0 | 0.063 | 2.5 ± 1.4 | 2.4 ± 1.3 | 0.195 |

| **Supplementary Table 3.** Correlation between the response to treatment and complications in all patients completing 6 and 12 months of treatment | | | | | | |
| --- | --- | --- | --- | --- | --- | --- |
| **All patients** | **After 6 months of treatment** | | | **After 12 months of treatment** | | |
|  | Non-Responders | Responders | P-value | Non-Responders | Responders | P-value |
|  | n=50 | n=47 |  | n=26 | n=42 |  |
| **Complications** | | | | | | |
| **Blisters and Vesicles** | 20 (40.0%) | 17 (36.2%) | 0.086 | 10 (38.5%) | 15 (35.7%) | 0.213 |
| **Lymphadenopathy** | 4 (8.0%) | 3 (6.4%) | 0.091 | 2 (7.7) | 4 (9.5) | 0.099 |
| **Vitiligo** | 3 (6.0%) | 2 (4.2%) | 0.121 | 1 (3.8) | 2 (4.8) | 0.418 |
| **Hyperpigmentation** | 2 (4.0%) | 3 (6.4%) | 0.093 | 1 (3.8) | 3 (7.1) | 0.103 |
